# Ocular Neurodegenerative Diseases: Interconnection between Retina and Cortical Areas

**DOI:** 10.3390/cells10092394

**Published:** 2021-09-12

**Authors:** Nicoletta Marchesi, Foroogh Fahmideh, Federica Boschi, Alessia Pascale, Annalisa Barbieri

**Affiliations:** Department of Drug Sciences, Pharmacology Section, University of Pavia, Viale Taramelli 14, 27100 Pavia, Italy; nicoletta.marchesi@unipv.it (N.M.); foroogh.fahmidehtavako01@universitadipavia.it (F.F.); federica.boschi@unipv.it (F.B.); alessia.pascale@unipv.it (A.P.)

**Keywords:** eye, SNC, retinal neurodegeneration, neurodegenerative diseases, age-related diseases

## Abstract

The possible interconnection between the eye and central nervous system (CNS) has been a topic of discussion for several years just based on fact that the eye is properly considered an extension of the brain. Both organs consist of neurons and derived from a neural tube. The visual process involves photoreceptors that receive light stimulus from the external environment and send it to retinal ganglionic cells (RGC), one of the cell types of which the retina is composed. The retina, the internal visual membrane of the eye, processes the visual stimuli in electric stimuli to transfer it to the brain, through the optic nerve. Retinal chronic progressive neurodegeneration, which may occur among the elderly, can lead to different disorders of the eye such as glaucoma, age-related macular degeneration (AMD), and diabetic retinopathy (DR). Mainly in the elderly population, but also among younger people, such ocular pathologies are the cause of irreversible blindness or impaired, reduced vision. Typical neurodegenerative diseases of the CSN are a group of pathologies with common characteristics and etiology not fully understood; some risk factors have been identified, but they are not enough to justify all the cases observed. Furthermore, several studies have shown that also ocular disorders present characteristics of neurodegenerative diseases and, on the other hand, CNS pathologies, i.e., Alzheimer disease (AD) and Parkinson disease (PD), which are causes of morbidity and mortality worldwide, show peculiar alterations at the ocular level. The knowledge of possible correlations could help to understand the mechanisms of onset. Moreover, the underlying mechanisms of these heterogeneous disorders are still debated. This review discusses the characteristics of the ocular illnesses, focusing on the relationship between the eye and the brain. A better comprehension could help in future new therapies, thus reducing or avoiding loss of vision and improve quality of life.

## 1. Introduction

**Figure 1:**
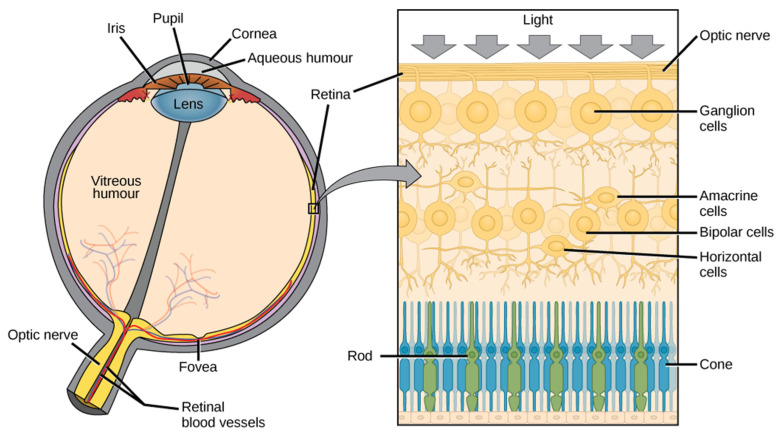
Anatomy of eye and retina. The different components and structures of the eye with the detail (in the right panel) of the composition of the retina.

The brain is linked to particular sense organs. We see from the eyes, and the information collected reaches specific neurons of visual cortex in the forebrain. The eye provides a unique window of the brain due to its special connection; the retina, photosensitive nerve tissue that covers the inner surface of the eye, is properly considered part of the brain [1,2,3]. The typical retina of a mammal contains several different types of neurons (Figure 1), each of which has its own morphology and specific function: The signals of the photoreceptors (rods and cones) are processed in cascade by groups of amacrine, bipolar, and horizontal cells that are in connection with the ganglion cells, responsible for transmitting information to the brain [4]. In detail, the light passes through the cornea, pupil, lens, and vitreous, and arrives to the retina, generating visual stimuli, which are transformed into electrical impulses and transported through the optic nerve to the brain. The brain interprets them by giving shape to images. Therefore, the retina is a functional unit of the central nervous system that converts a light signal into a nerve impulse, being physically connected to the brain via axons of the optic nerve. The complex synaptic connections that underlie the visual system have long been known [5,6,7]. Despite their different morphology, retinal ganglionic cells (RGCs) and neurons are anatomically similar; both are made up of a cell body, dendrites, and axons. RGCs’ axons form the optic nerve (Figure 1) that is covered with the myelin and with meninges, like all other nerve fiber tracts. The central nervous system and the eye are protected sites. The eye has the blood–retinal barrier (BRB) and the CNS has the blood–brain barrier (BBB). These barriers are quite similar, being both composed of non-fenestrated endothelial cells connected by tight junctions [3]. The endothelial cells that form the BBB and BRB are able to provide oxygen and glucose in adequate concentrations for neuronal function, while they prevent the flux of other molecules and cells in order to protect the neuronal environment [8]. Both the eye and the brain have limited capacities for regeneration. Apart from the presence of a limited number of progenitors, nerve cells do not replicate. Hence, degeneration as well as immune-mediated inflammation can induce irreversible damage to neurons, atrophic alterations like those present in neurodegenerative diseases, typical of the central nervous system, or blindness.

The retinal tissues consist of neurons, vascular tissue, and glial cells, which interact each other, resulting in a neurovascular system. In particular, the retina is structurally made up of two overlapping sheets: one external in contact with the choroid, pigment epithelium, and the other one internal in relation to the vitreous body, sensory retina. The pigment epithelium maintains the external blood–ocular barrier. Retinal blood vessels maintain the internal blood–ocular barrier. 

The breakdown of the blood–retinal barrier (BRB) is considered pathognomonic for the development of retinopathy [9]. Moreover, degeneration of specific retinal neurons in several ocular diseases (i.e., glaucoma, age-related macular degeneration (AMD), diabetic retinopathy (DR), and retinitis pigmentosa (RP)) is the leading cause of irreversible blindness [3,10]. Indeed, in the industrialized world, the most frequent causes of blindness are eye neuropathology diseases. Prevalence varies with age. While age-related macular degeneration is the most frequent cause in old age, younger people are more often affected by diabetic retinopathy.

Briefly, glaucoma is characterized by progressive optic nerve degeneration and can be considered a neurodegenerative disorder of both the eye and the brain [11,12]. Age-related macular degeneration is a common eye disease in the macula, the part of retina responsible for sharp, central vision. It causes visual recognition difficulties. It is also associated with higher rates of cognitive decline in late life and higher risk of dementia [13,14]. Neurodegeneration plays a significant role in the complex pathology of diabetic retinopathy. Evidence suggests the onset of neurodegeneration occurs early on in such disease [15,16]. Retinitis pigmentosa is a retinal dystrophy. It is characterized by the progressive degeneration and death of photoreceptors, resulting in an initial loss of night vision and a progressive constriction of the visual field [17,18]. Retinitis pigmentosa does not have similarities with a particular disease of the CNS but there is evidence that it is correlated with significant reductions in gray matter volume, mainly in the occipital cortex of RP patients [19].

Nowadays, few drugs are approved for the treatment of the abovementioned eye diseases [20]. Generally, the therapies are still limited to symptomatic actions. A summary of the main drugs currently in use in therapy is reported in Table 1.

In order to achieve therapeutic benefits in ocular impairments, focused on tissue repair or regeneration, several strategies such as gene therapy, stem cell therapy, and target discovery through genomic research represent significant promise [21]. Currently, there is no therapy to modify the disease-associated degenerative changes and no effective treatment to reverse the loss of vision when photoreceptors degenerate or lose their ability to transmit the visual stimuli to the brain or when retinal ganglionic cells die. In recent decades, experimental evidence underlines the approach to treat blinding diseases through regenerative medicine [10,22]. Some innovative ocular therapies, based on a variety of transplantable products and novel drug-delivery technologies including nanoparticles, nanomicelles, and liposomes, should be able to revolutionize treatment of numerous blinding disorders [23]. 

However, the strong link between the eye and the central nervous system is supported by evidence that the ocular alterations existing in various neurodegenerative pathologies of the CNS and visual manifestations sometimes precede central symptoms. Moreover, the retina is a CNS compartment that can be easily analyzed with optical techniques, such as, for example, the optical coherence tomography (OCT), so retinal changes may reflect the pathological features in the brain early in the disease processes [24,25].

Moreover, several etiological factors such as oxidative stress, neuroinflammation, proteolytic degradation, dysregulation of ocular hemodynamic parameters, trans-synaptic degenerative changes, genetic causes, and aberrant cellular signaling are involved in neurodegeneration and cell loss associated with both CNS and retina disorders [26].

**Table 1:** Some drugs currently used in major ocular diseases.

Diseases	Therapeutic Category	Drugs	Route of Drug Administration	Effect/Mechanism of Action	References
**Glaucoma**	Prostaglandin analogue	Latanoprost Bimatoprost Travoprost	Topical instillation (drops)	Reduction of intraocular pressure (IOP)/Increase uveoscleral outflow	[27,28]
	Protaglandin/Rho-kinase transporter inhibitor association	Latanoprost/Netarsudil	Topical instillation (drops)	Increase of the trabecular outflow/Decreasing the aqueous production/Decrease the episcleral venous pressure	[28,29,30,31]
	Rho-kinase transporter inhibitor	Netarsudil	Topical instillation (drops)	Reduction of intraocular pressure (IOP)/Increase of the trabecular outflow	[32]
	β-receptor antagonists	Timolol Betaxolol Levobunolol Carteolol Metipranolol	Topical instillation (drops)	Reduction of intraocular pressure (IOP)/Decrease aqueous humor production	[28,33,34]
	α2-receptor agonists	Brimonidine Apraclonidine	Topical instillation (drops)	Reduction of intraocular pressure (IOP)/Decrease aqueous humor production	[28,33,34,35]
	Carbonic anhydrase inhibitors	Brinzolamide Dorzolamide	Topical instillation (drops)	Reduction of intraocular pressure (IOP)/Decrease aqueous humor production and increase uveoscleral outflow	[28,33,34,36]
	Cholinergic receptor agonists	Pilocarpine Carbachol	Topical instillation (drops)	Reduction of intraocular pressure (IOP)/Increase trabecular outflow	[28,34]
**AMD**	Anti-VEGF	Ranibizumab Aflibercept Pegaptanib Conbercept Brolucizumab	Intravitreal injection	Reduction of new blood vessel growth/Inhibition of the biological activity of VEGF	[31,37,38,39]
	Photodynamic therapy	Verteporfin	Intravenous	Elimination of the abnormal blood vessels in wet-form macular degeneration	[39,40]
**DR**	Anti-VEGF	Ranibizumab Aflibercept Bevacizumab	Intravitreal injection	Reduction of new blood vessel growth/Inhibition of the biological activity of VEGF	[16,41]
**RP**	Supplements/Vitamin	Vitamin A Omega 3 (DHA) Lutein	Topical instillation/oral	Improve photoreceptor metabolism, slowing its death by apoptosis	[42,43,44,45]

AMD: Age-Related Macular Degeneration; DR: Diabetic Retinopathy; RP: Retinitis Pigmentosa; IOP: Intraocular Pressure; VEGF: Vascular Endothelium Grown Factor; DHA: Docosahexaenoic Acid.

## 2. CNS and Eye Neurodegeneration

**Figure 2:**
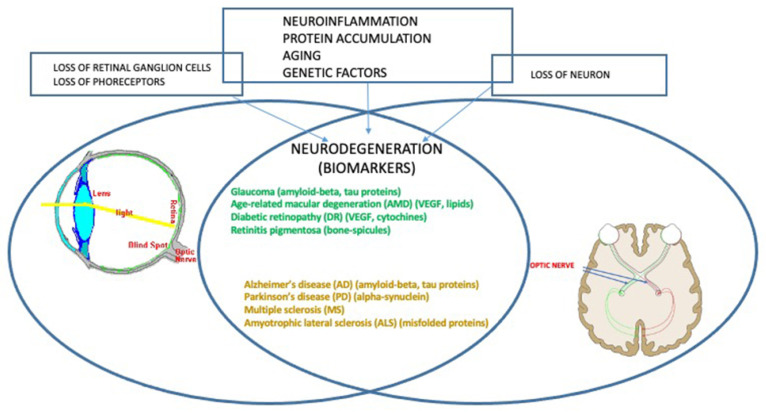
Central nervous system and ocular neuropathologies. The CNS and the eye share the same factors in the etiophatology of disease. Neurodegeneration is common, as are some biomarkers, reported in parentheses for each pathology.

Neurodegenerative CNS pathologies are debilitating and quite untreatable diseases that involve morphologic alterations and progressive loss of function of neurons, thus causing progressive degeneration and/or death of nerve cells. They include both movement disorders (so-called ataxias) or mental disfunction (so-called dementias). CNS diseases involve neurons, glial cells, and the vascular system, even if only the neurons suffer progressive damage. The communication between the cells occurs early; then the entire cellular structure is compromised up to death. Genetic factors are also involved in the etiopathogenesis of some illnesses; genetic influence increases the chances of developing neurodegenerative diseases [46,47] (Figure 2). The most known, widespread, and common illnesses among the elderly population are Alzheimer disease and Parkinson disease, affecting worldwide more than 30 million and 5 million subjects, respectively [48,49]. Some evidence highlights that neurodegenerative processes involve both the central nervous system and the retina, being the anterograde (postsynaptic neurons) and retrograde (presynaptic neurons) trans-synaptic neurodegeneration, caused by retinal ganglion cells’ death, the main mechanism involved [50]. Such atrophic neural alterations involve neurons and axons as a result of injury of the cells with which they are in communication, causing an interruption of the synaptic stimulus [51]. Therefore, trans-synaptic degeneration is a process that spreads damage from the initial site to neuronal projections. Such a trans-synaptic degeneration has been long proven in the motor system and cerebellar pathways; only over the last decades, the presence of retrograde trans-synaptic degeneration has been highlighted in the human visual system, with particular relation to glaucoma [52,53,54]. It contributes to visual impairment observed in association with other various diseases, including, for example, multiple sclerosis [55]. Moreover, CNS pathologies display ocular manifestations due to direct degeneration of the visual pathways [56], often related to a direct injury to the optic nerve and/or retinal ganglion cells [57]. Alzheimer disease and glaucoma, both being diseases of the elderly, have several epidemiological and histological overlaps in pathogenesis [58]. Among the etiological factors, the neuroinflammatory response is considered of crucial importance in the major neurodegenerative diseases of the CNS related to age; such inflammatory response in the brain can occur in the retina as it represents an extension of the brain. Nevertheless, microglial cells, the immunocompetent cells of the CNS, are key factors in these neurodegenerative lesions as they respond to injury and degeneration with morphological changes, proliferation, migration, and inflammatory cytokine production [59]. Furthermore, a number of microglia can rapidly increase under pathological conditions, such as inflammation/neuroinflammation, and, therefore, they are implicated in the initiation and progression of several neurological disorders; they represent a common hallmark of various retinal degenerative and inflammatory diseases [60,61]. 

For some time, science has been studying if retinal neurodegeneration is predictive for Alzheimer disease (AD) and Parkinson disease (PD). In patients with AD or PD, several studies have shown changes in the nerve fibers of the retina [2]. It was noted that subjects with preclinical AD showed retinal microvascular and structural alterations. Venous narrowing and reduced blood flow have been especially determined [62]. PD is also associated with retinal thinning [63,64], which, as a consequence, is associated with reduced retinal blood flow. Visual abnormalities are prominent in AD, and most of them are due to RGC loss and are believed to develop before cognitive impairment [14]. In particular, Alzheimer disease is characterized by the accumulation in the brain parenchyma of extracellular amyloid-beta (Aβ) peptide aggregates, by intracellular deposits of hyperphosphorylated tau protein, by neurodegeneration, and glial activation). However, these changes occur in the brain long before cognitive deficits. The challenge is to be able to recognize these disorders before clinical symptoms [65]. Recent evidence from human samples and mouse models indicates the possibility of detecting protein aggregates and other distinctive pathological hallmarks in the retina, providing the way for rapid non-invasive detection of Alzheimer disease biomarkers [66]. Generally, these biomarkers are detected either through cerebrospinal fluid analysis, brain imaging, or post-mortem. Given that the eye possesses neural and vascular similarities to the brain, it is now strongly underlined that the retina is a direct window through which it is easier to possibly monitor the neurodegeneration processes linked to Alzheimer disease [14,48,67]. Interestingly, retinal Aβ levels, which reflect those of the brain, appear to become a promising opportunity for early detection of AD-related cerebral changes and cognitive decline [48]. Currently, all the ocular biomarkers (i.e., studied with detection of Aβ-related retina changes, PET (positron emission tomography) imaging, OCT (optical coherence tomography) and OTC angiography, and cerebral spinal fluid molecules) are considered in a promising way as a means to improve, understand, and monitor adequate AD and other neurodegenerative diseases’ therapies [68].

It is well known that Parkinson disease is characterized by the loss of dopaminergic neurons in the *substantia nigra*. Furthermore, it is also established that, before degeneration, in dopaminergic neurons it is evident that proteins accumulate, one of which in particular, alpha synuclein, seems to play a fundamental role. In fact, as the disease progresses, a great amount of this protein (known as Lewy bodies) are found increasingly widespread [69]. Parkinson disease patients show very early vision defects and alpha-synuclein accumulations also in the retina [70]. Furthermore, mutations that induce an increase in the expression of this protein lead to Parkinson disease, demonstrating that this protein seems to play a crucial role in the pathogenesis. PD is usually diagnosed on the presence of several motor symptoms, such as tremors, muscle stiffness, and balance problems. PD is also associated with several non-motor symptoms including disorders of mood, such as apathy, anhedonia and depression, cognitive dysfunction in the form of working memory deficits, and complex behavioral disorders [71]. However, motor symptoms develop after prolonged progression, with significant damage to the dopaminergic neurons. Interestingly, it was demonstrated that in PD there is also a thinning of the retinal walls and retinal microvasculature alterations [64], so that, this ocular damage could represent an early non-motor symptom of the disease. Therefore, Parkinson disease progression is also associated with the structural changes of the retinal nerve fiber layer; in fact, greater changes at this level as well as macular thickness were found in patients with PD compared to controls [72]. These axonal alterations caused by PD can be detected using optical coherence tomography, an imaging technique developed to evaluate retinal disease, and these special measurements are usefully considered as biomarkers of PD progression [73]. However, not only AD and PD, but also other neurodegenerative CNS diseases are related to ocular damages. 

Optic neuritis can be an early sign of multiple sclerosis (MS), an autoimmune demyelinating and neurodegenerative disease of the central nervous system. Pathogenic mechanisms include inflammation by T- and B-lymphocytes and cells of innate immunity as well as oxidative stress; several other factors that lead to neurodegeneration include microglia activation, chronic oxidative injury, and accumulation of mitochondrial damage in axons [74,75]. Inflammation of the optic nerve, a condition known as optic neuritis, is one of the most frequent clinical manifestations and it can be an early sign of MS. The consequences are different vision disturbances such as a decrease in monocular visual acuity, often associated with pain, oscillopsia, linked to the presence of nystagmus, and diplopia, i.e., double vision caused by imperfect alignment of the eyeballs usually due to an injury to the oculomotor system. Patients with multiple sclerosis have also shown a reduction in the optic nerve perfusion and in the thickness of the retinal nerve fiber layer compared to healthy subjects. Recent evidence demonstrated that OCT and OCT-angiography images reflect the loss of retinal ganglion cells and axonal damage due to MS [76].

Additionally, amyotrophic lateral sclerosis (ALS) affects the neurons of the central nervous system, in particular, the spinal and cortical motor neurons. It is a fatal, progressive, degenerative pathology involving loss of the first motor neurons located in the brain and the second motor neurons located in the brain stem and spinal cord. These events lead to the loss of control of the muscles responsible for movement. In up to 50% of the affected population, there are other extra-motor manifestations such as changes in behavior, executive dysfunction, and language disturbances, and these problems are so severe to meet the clinical requirements of frontotemporal dementia in 10%–15% of patients [77]. Underlying the pathology, several molecular mechanisms are involved, such as excitotoxicity, mitochondrial disorders, alterations in axonal transport, oxidative stress, accumulation of misfolded proteins, and neuroinflammation, in addition to genetic factors [78]. Recently, it has been shown that it also affects the visual system; although at present ophthalmic complications are not considered as a classic symptom of ALS, recent evidence underlines that retinal changes such as thinning, axonal degeneration, and protein inclusion have been found in many patients [79]. Therefore, even in these circumstances, the retinal conditions are being proposed as a possible biomarker of ALS.

## 3. Ocular Neurodegenerative Diseases 

### 3.1. Glaucoma

Glaucoma is currently one of the most common causes of irreversible visual impairment and blindness in the world [80]; it includes a group of heterogeneous eye diseases, with closed-angle glaucoma and open-angle glaucoma the two main types. Generally, glaucoma is due to the increase in the internal pressure of the eye, that is, the intraocular pressure (IOP), which irreparably damages neurons; in some cases the reduction of the blood supply to the optic nerve, which cause loss of visual field, is involved [54,81]. In recent years, the literature argues in favor of the fact that glaucoma is a widespread neurodegenerative disease involving the CNS, as the correlation is strong between the dysfunction and death of CNS neurons with retinal ones. Moreover, neurodegenerative pathways that contribute to transynaptic neurodegeneration in AD, as well as in other CNS diseases, might also be similar to those in neurodegeneration correlated to glaucoma [11,82]. Retinal ganglion cell damage is a characteristic of both glaucoma and AD, along with discovery of amyloid-beta and tau protein deposition, known to be pathognomonic of AD, in the retina and aqueous humor of the eye [58]. In particular, primary open-angle glaucoma (POAG), the most common type, is characterized by slow, progressive, degeneration of retinal ganglion cells and their axons in the optic nerve, leading to visual field defects [83]. Intraocular pressure (IOP) is considered a major risk factor for the development of POAG, and the modified optic nerve head is the site of initial damage. However, elevated IOP is not present in all types of POAG, and in normal-tension glaucoma IOP is not elevated, so other risk factors are likely involved in the optic neuropathy. Literature evidence provides that the pressure and composition of the cerebro-spinal fluid (CSF) surrounding the optic nerve may have critical involvement in the pathogenesis of glaucoma [83]. In this regard, the presence of the glymphatic system was described. This particular system is a brain-wide paravascular pathway for CSF–interstitial fluid exchange that facilitates clearance of interstitial solutes, including amyloid-beta, from the brain. If the glymphatic system does not operate properly, amyloid-beta brain accumulation occurs in AD. In the same way, the glymphatic system may also have potential clinical relevance for the understanding of glaucoma. Aβ accumulation may be implicated in the development of retinal ganglionic cells’ apoptosis. Recent studies indicated that accumulation of amyloid-beta, which is associated with the progression of Alzheimer disease, may also be responsible for retinal ganglion cell death in glaucoma, so the neurodegenerative processes in glaucoma could share, at least in part, a common mechanism with Alzheimer disease [83]. Interestingly, literature data, although derived from animal studies, found time-dependent expressions and localization of Aβ in the retina as well as in the optic nerve head after chronic IOP increase seen in glaucoma [84]. Moreover, nowadays, it is well established that glaucoma leads to ganglion cell death through several other mechanisms including oxidative stress, neuroinflammation, and mitochondrial dysfunction [85,86]. Retinal ganglion cells and optic nerve fibers are particularly rich in mitochondria, necessary organelles to produce energy for nerve conduction. The reduction in energy production and the increase in the production of free radicals at the mitochondrial level are to be considered as potential additional mechanisms in the etiopathogenesis of glaucoma. Definitely, the identification of cellular mechanisms and molecular pathways related to retinal ganglion cell death is the first step toward the discovery of new therapeutic strategies to control glaucoma [87,88].

### 3.2. Age-Related Macular Degeneration

Age-related Macular Degeneration (AMD) is an ocular pathology that involves the central area of the retina, the so-called macula, causing an irreversible reduction in distinct vision, and it is one of the leading causes of blindness in developed countries. AMD is classified into a dry form, with about 80% of incidence in the population affected, and a wet form or neovascular form, with about 20% of incidence. In particular, in dry age-related macular degeneration, characteristic lesions, called drusen, appear. These are accumulations of cellular waste that can be reabsorbed or calcified. In wet macular degeneration, in addition to drusen, there is the anomalous formation of new vessels under the retina, responsible for the exudative evolution of macular degeneration [89,90]. Therefore, localized sclerosis under the retina, the accumulation of lipids, and alterations in the metabolism of the retinal pigment epithelium (RPE) contribute to the macular degenerative process [91]. Under these conditions, the physiological metabolism of the retina is prevented. Moreover, retinal hypoxia may induce an upregulation of VEGF by the RPE and thus promote the growth of abnormal vessels from choroid, with VEGF being the main factor related to ocular neovascularization [92,93]. Furthermore, the RPE is crucial for the maintenance of photoreceptor cells as it promotes a physiological vascular environment. In particular, RPE keeps retinal nerve tissue healthy by secreting hormones, transporting molecules, eliminating dead cells, and modulating immune factors. The RPE is responsible for the transport of nutrients, ions, and water. It absorbs light and protects the retina from photooxidation; in addition, it is responsible for stabilizing the concentration of ions in the subretinal space to keep the photoreceptors excitable. To maintain RPE homeostasis and function, a particular molecular network is necessary, with microRNAs being indispensable components [94].

With aging, several modifications occur in the RPE cells as a result of their altered capacity for removing residual substances, leading to a further damage in the pathogenesis of AMD. The main risk factor for AMD is age, but family history, female sex, smoking, and high blood pressure can somehow contribute; among these, several studies suggest that smoking is the main oxidative stress factor [20]. Nevertheless, different oxidative damage, such as light exposure or inflammation that affect the retina, has been strongly linked with AMD [37]. Globally considered together, oxidative stress and mitochondrial damage in the retinal pigment epithelium may play an important role in the pathogenesis of age-related macular degeneration [95]. It is well known that mitochondrial dysfunction has been associated with aging, as well as with several age-related diseases, such as Alzheimer and Parkinson diseases, suggesting that ocular and CNS neuropathologies share more than one biochemical mechanism. Moreover, AMD also is associated with non-visual impairment such as phonemic verbal fluency, verbal memory, establishment of cognitive decline during life, and higher risk of dementia [13,96]. Once again, it is emphasized how cognitive impairment, as well as visual impairment, is common and superimposed among older adults. 

### 3.3. Diabetic Retinopathy

Diabetic retinopathy (DR) is a complication of diabetes that affects the eyes, causing severe visual impairment. It is induced by damage to the blood vessels in the light-sensitive part of the eye, the retina, with the vasculopathy being the main involved pathophysiologic mechanism [15]. It can develop in subjects affected by both type 1 and type 2 diabetes. There are two types of retinopathies. The first is the early diabetic retinopathy, also known as nonproliferative diabetic retinopathy (NPDR). As the disease progresses, the walls of the blood vessels weaken and are subject to microaneurysms, small swellings that, when damaged, lead to bleeding. Then there is the risk of an accumulation of fluids, i.e., formation of edema, in the macula, which cause reduced vision. The second type is proliferative or advanced diabetic retinopathy (PDR). It is the most serious type because it coincides with the abnormal growth of new blood vessels damaging the retina. Diabetes is, in fact, associated with a growth of weak blood vessels, more prone to rupture, or smaller vessels, and this leads to a lower oxygen transport capacity to the retinal tissues. As a result, new vessels are stimulated by the formation of ischemic areas in the retina. In fact, retinal microvascular disease is an early compromission, induced by low-grade, persistent leukocyte activation, which causes repeated episodes of capillary occlusion and progressive retinal ischemia [97]. This situation can induce detachment of the retina or an abnormal flow of fluid into the eye, causing glaucoma. The underlying molecular mechanisms associated with vascular dysfunction, especially endothelial dysfunction, in DR are multifactorial. Chronic inflammation, oxidative stress, leukostasis, dysregulated growth factors and cytokines, and disruption of peroxisome proliferator-activated receptor-γ are mainly involved [98]. Diabetic retinopathy is prevalent in around 35% of patients with diabetes. The disease progresses slowly, causing damages that become progressively irreversible. Unfortunately, treatment options are limited. As therapeutic approaches, photocoagulation of the ischemic areas of the retina to stabilized blood vessels, intravitreal injections with VEGF-inhibitory agents or corticosteroids, and ocular surgery can be applied [99]. It is worth noting that anti-VEGF agents used in clinical practice, such as ranibizumab, bevacizumab, and aflibercept, are considerably different in terms of molecular interactions when they bind with VEGF [100]; therefore, characterization of such features can improve the design of novel biological drugs potentially useful in clinical practice. Recent findings hypothesize that retinal neurodegeneration represents a critical, early component of DR. It occurs prior to the vascular changes classically associated with DR and contributes to disease pathogenesis [101,102]. In the retina, neurons, glia, and vasculature form the blood–retinal barrier (BRB), which functions as the maintenance of energy, homeostasis, and neurotransmitter regulation. In the progression of diabetes, the BRB is damaged early and its breakdown is sustained by RPE secretion of different factors, among which the main ones are vascular endothelial growth factor (VEGF) and proinflammatory cytokines (i.e., TNF-α, IL-6, IL-1β) [103]. It is interesting to note that VEGF may act as a negative regulator of pericyte function, with these cells being involved in early BRB abnormalities in diabetic retinopathy [104]. During progression of DR, the retina is infiltrated by the above mentioned secreted factors’ cells and serum proteins, further damaging blood vessels and neurons. Moreover, in addition to vascular damage and the loss of BRB integrity, other neurodegenerative changes occur in the retina such as apoptosis, glial cell reactivity, microglial activation, and altered glutamate metabolism that could prove some of the functional deficits in vision [101,105]. Additionally, to point out the neurodegeneration, clinical evidence indicates CNS lesions in patients with diabetic retinopathy; detection of small punctate white matter lesions in the brain and cortical atrophy in some regions suggests that there is an association between retinopathy and brain tissue damage [106]. Other studies highlight that diabetes-induced retinal neurodegeneration and brain neurodegenerative diseases share common pathogenic pathways. Indeed, DR patients might exhibit abnormalities in the central nervous system, often showing impaired cognition and increased risks of dementia as well as Alzheimer disease [107].

### 3.4. Retinitis Pigmentosa

Retinitis pigmentosa (RP) is an inherited retinal dystrophy leading to progressive loss of the photoreceptors and retinal pigment epithelium and resulting in blindness usually after several decades [17,108,109]. Usually, it is bilateral, but some evidence reports of unilateral eye involvement with RP. It affects approximately one subject in 5000 worldwide, making RP one of the most common inherited diseases of the retina [110]. Generally, degeneration of rod photoreceptors, the cells controlling night vision, precedes and exceeds cone degeneration, as a majority of RP genetic mutations affect rods selectively. Early symptoms of retinitis pigmentosa include impaired night vision and peripheral vision [109]. The main clinical hallmarks consist of bone-spicule deposits, waxy optic disc, and shrinked retinal vessels. As previously pointed out, retinitis pigmentosa does not share alterations with neurodegenerative diseases of the CNS; it is linked with reduction of white matter volume in the brain, as seen in RP patients [19]. 

There is no definitive therapy. In some cases it is possible to slow down the degenerative process with strategies such as the administration of vitamins, and protection from sunlight and combined approaches, such as gene-replacement therapy, may be useful to slow photoreceptor cell death [111,112]. Different RP gene mutations are the basis of alterations in molecular mechanisms such as phototransduction cascade, vitamin A metabolism, interactive cell–cell signaling or synaptic interaction, and intron splicing of RNA [113]. Moreover, previous studies revealed that an insufficiency of the ubiquitin-proteasome system (UPS) to process misfolded proteins in affected photoreceptor cells could be involved [114]. Impairments of UPS function in the central nervous system underlie an increasing number of genetic diseases, many of which affect the retina [115].

## 4. Conclusions

As the prevalence of neurological diseases increases dramatically with age and the aging population increases, neurodegenerative diseases could have an ever-increasing impact on people’s quality of life. Early diagnosis and optimal follow-up are critical for better disease management and for delaying progression and disability. Growing evidence suggests that the eye is like the brain: Both organs can suffer the effects of time and they could be affected by neurodegeneration. While brain damage occurs mainly in the form of cognitive diseases, such as Alzheimer or Parkinson, neurodegeneration can present itself in the form of glaucoma in the eyes. The retina and optic nerve are an embryological extension of the brain tissue, and the retina provides a unique opportunity to evaluate the alterations caused by neurological diseases, showing a cellular composition similar to that of brain tissue. Through optical coherence tomography (OCT), a high-resolution technology, it is possible to detect alterations in clinical conditions. Therefore, a measurement of changes in intraretinal layer thickness is a reliable signal linked to axonal loss or related neuroinflammation of neurodegenerative pathologies. Early signs of retinal damage are present in Parkinson and Alzheimer diseases as well as in multiple sclerosis. There is increasing evidence that beta-amyloid is a factor involved in the development of ganglion cell apoptosis in glaucoma; preclinical studies demonstrate that retinal ganglion cells, subjected to a chronic increase in intraocular pressure, show abnormal processing of a precursor protein of beta-amyloid, suggesting a correlation between AD and glaucoma [11]. Accumulations of alpha-synuclein in the brain present in Parkinson disease affect dopaminergic neurons, leading to the development of motor symptoms. If high concentrations of this protein are present in the retina, it leads to the death of amacrine cells that contain dopamine, leading to a reduction in visual acuity [116]. Moreover, cerebro-spinal fluid circulatory failure contributes to the development of glaucoma. AMD is a reduction of visual function related to the aging process of the eye: The macula, containing numerous photoreceptors, alters until it loses its characteristics. This phenomenon is due to retinal cell degeneration and death. Moreover, AMD and AD share the same biomarkers; in AMD there is also evidence of protein misfolding disease similar to Alzheimer disease. Recent studies show that differential expression of miRNAs (miR-9, miR-23a, miR-27a, miR-34a, miR-146a, miR-155) has been found to be dysregulated both in AMD and AD [117]. Current evidence suggests that neurodegeneration of the retina is a critical component of diabetic retinopathy, in addition to the damage to the blood vessels of the ocular tissue. Retinitis pigmentosa is a retinal dystrophy characterized by the gradual loss of photoreceptors and dysfunction of the pigment epithelium. This pathological context put into evidence that the retina progressively reduces its ability to transmit visual information to the brain via the optic nerve. A great importance lies in the studies of those measurable substances within the body, called biomarkers, which monitor the development of the disease and the effectiveness of potential drugs. Since the neurodegenerative process is already biologically advanced by the time the symptoms appear, having biomarkers available in the preclinical phase, to signal the pathological process in progress, is essential to obtain effective therapies, even more if the biomarkers are sensitive to therapeutic treatments.

## Data Availability

Not applicable.
